# Parisian Doings

**Published:** 1860-05

**Authors:** 


PARISIAN DOINGS.—Dr. Robert Battey, of Georgia, a correspondent of the *Atlanta Medical and Surgical Journal*, writing from Paris, under date of January, 1860, states that Jobert totally ignores the American operation for the cure of vesico-vaginal fistule, and relies upon a process of his own. The surgeon of the Hôtel-Dieu is described as a haughty, ill-natured man, of middle age, formerly handsome, and still good looking. "If you chance, momentarily, to obstruct his light, he demands, in a stern tone, 'Get out of my *day!*' and lectures you soundly for your impudence. Woe to the unlucky interne who gets in his light or jostles his elbow." * * * "M. Jobert, as he sits at his operating table, and growls, and cuts, and scolds, does naturally not make a very favorable impression upon strangers; but it must be conceded that he is an expert operator in general surgery."

At Laraboisiere, Chassaignac treats fractures, after the subsidence of inflammation, nearly altogether with the plaster bandage. A roller is first applied, and then thickly smeared over with a cream of plaster of Paris, which thus forms a firm, immovable support for the limb.

"I was surprised," says Dr. Battey, "to hear from an eminent Parisian surgeon the caustic remark, 'M. Ricord is but a syphilitic fungus grafted upon the Hospital Midi!'" The French syphilographer has evidently got enemies.

The latest novelty in Paris is the attempt, on the part of two German physicians, Moncel and Ruhmkorff, to teach French surgeons how to illuminate the mucous outlets of the body by the aid of luminous tubes, transmitting a current of electric light. The subject is now before the Academy, to be reported upon by M. Cl. Bernard, the eminent physiologist.

